# *De Novo* Unbalanced Translocations in Prader-Willi and Angelman Syndrome Might Be the Reciprocal Product of inv dup(15)s

**DOI:** 10.1371/journal.pone.0039180

**Published:** 2012-06-14

**Authors:** Elena Rossi, Roberto Giorda, Maria Clara Bonaglia, Stefania Di Candia, Elena Grechi, Adriana Franzese, Fiorenza Soli, Francesca Rivieri, Maria Grazia Patricelli, Donatella Saccilotto, Aldo Bonfante, Sabrina Giglio, Silvana Beri, Mariano Rocchi, Orsetta Zuffardi

**Affiliations:** 1 Medical Genetics, University of Pavia, Pavia, Italy; 2 Scientific Institute Eugenio Medea, Bosisio Parini, Lecco, Italy; 3 Department of Pediatrics, San Raffaele Scientific Institute, Vita-Salute San Raffaele University, Milan, Italy; 4 Department of Pediatrics, University Federico II, Naples, Italy; 5 Medical Genetics Department, APSS Trento, Trento, Italy; 6 Biologia Molecolare Clinica e Citogenetica, Diagnostica e Ricerca, San Raffaele SPA, Milan, Italy; 7 Genetica Medica, Ospedale Civile, Bassano del Grappa, Italy; 8 Medical Genetics Unit, Meyer Children's Hospital, University of Firenze, Firenze, Italy; 9 Department of Biology, University of Bari, Bari, Italy; 10 IRCCS “C. Mondino National Neurological Institute” Foundation, Pavia, Italy; Institut Jacques Monod, France

## Abstract

The 15q11-q13 region is characterized by high instability, caused by the presence of several paralogous segmental duplications. Although most mechanisms dealing with cryptic deletions and amplifications have been at least partly characterized, little is known about the rare translocations involving this region. We characterized at the molecular level five unbalanced translocations, including a jumping one, having most of 15q transposed to the end of another chromosome, whereas the der(15)(pter->q11-q13) was missing. Imbalances were associated either with Prader-Willi or Angelman syndrome. Array-CGH demonstrated the absence of any copy number changes in the recipient chromosome in three cases, while one carried a cryptic terminal deletion and another a large terminal deletion, already diagnosed by classical cytogenetics. We cloned the breakpoint junctions in two cases, whereas cloning was impaired by complex regional genomic architecture and mosaicism in the others. Our results strongly indicate that some of our translocations originated through a prezygotic/postzygotic two-hit mechanism starting with the formation of an acentric 15qter->q1::q1->qter representing the reciprocal product of the inv dup(15) supernumerary marker chromosome. An embryo with such an acentric chromosome plus a normal chromosome 15 inherited from the other parent could survive only if partial trisomy 15 rescue would occur through elimination of part of the acentric chromosome, stabilization of the remaining portion with telomere capture, and formation of a derivative chromosome. All these events likely do not happen concurrently in a single cell but are rather the result of successive stabilization attempts occurring in different cells of which only the fittest will finally survive. Accordingly, jumping translocations might represent successful rescue attempts in different cells rather than transfer of the same 15q portion to different chromosomes. We also hypothesize that neocentromerization of the original acentric chromosome during early embryogenesis may be required to avoid its loss before cell survival is finally assured.

## Introduction

A variety of different structural rearrangements involving the proximal 15q have breakpoints mapping to the segmental duplication blocks (BP1-BP5) [1], [2] present in this genomic region. Although the mechanisms dealing with cryptic deletions and amplifications, including inv dup(15)s, have been partly characterized [1], [3]–[9], less is known about the translocations involving this region. Particularly interesting are the *de novo* unbalanced translocations of the chromosome 15 long arm to the telomeric region of another chromosome, characterized by 45 chromosomes, monosomy of 15p and of the proximal 15q imprinted region. In 2007, Mignon-Ravix et al. [10] demonstrated, by FISH analysis, that in four of eight patients with this type of translocations, either de novo or inherited, breakpoints clustered in an interval of about 460 kb at 15q14, distal to BP5, between BACs RP11-64O3 and RP11-150L8. They suggested this region could be a specific hotspot for unbalanced translocations involving the proximal 15q region. To test this hypothesis, we characterized at the molecular level five such unbalanced translocations, four *de novo* and one inherited, all resulting in the loss of the short arm and proximal long arm of chromosome 15. In order to obtain new insight in the mechanisms generating de novo unbalanced translocations involving the 15q1 region, we investigated whether the breakpoints of these translocations, usually associated with PWS or AS phenotype, depending on their parental origin, occurred as a consequence of the specific genomic architecture of this region.

## Results

We collected five cases with unbalanced translocation, first detected by karyotype analysis (Table 1). In one subject (case 5), the unbalanced translocation was in mosaic with a cell line containing the *de novo* balanced form of the same translocation. Three translocations were de novo, while the one in case 4 was inherited from the mother (Table 1).

**Table 1:** Phenotype, karyotype and molecular characterization of the five cases with unbalanced translocations.

Case	Phenotype	Karyotype	Parental Origin	Chr 15 Breakpoint interval	Recipient chromosome Breakpoint interval
1	PWS	45, XX, der(5)t(5;15)(q35;q11.2),-15	Unknown	Del/Dup:26220595-26234595, within a LINE repeat. Dup/N:27106557-27108882	Chr5:180615093-180615701
2	PWS	45, XX, der(18)t(15;18)(q13;q23), -15[97]/45, X, der(X), t(X;15) (q28;q13),-15[3] *	*De novo*	22838840-22846406, within a 7.5 Kb cluster of Alu and LINE repeats	Chr18: no copy number changes detected at the breakpoints. ChrX: no copy number changes detected at the breakpoints.
3	PWS	45, XY, der(6)t(6;15)(p25.3;q13),-15	*De novo*	25941268-25941852, within the *OCA2* gene	Chr6: no copy number changes detected at the breakpoints
4	AS	45, XX, der(9)t(9;15)(p24;q13),-15	Maternal	23579790–23580274, inside intron 1 of the *ATP10A* gene	Chr9:4074000-4086000
5	PWS	Mos46, XX, t(8;15)(p23.3;q14)[80]/45, XX, der(8)t(8;15)(p23.3;q14),-15[20]	*De novo*	30944015-30952913, in the 5′ upstream sequence of the *FMN1* gene	Chr8: no copy number changes detected at the breakpoints

* The minor cell line has been confirmed, by classical cytogenetics, in fibroblasts, with a similar mosaicism percentage (45, XX, der(15;18)(q13;q23)[83]-15/45, X, der (X;15)(q28;q13),-15[3]*).

Imbalances were associated with PWS in four patients and AS in one, indicating that in four cases the 15 deletion occurred in the paternal chromosome and in one case in the maternal one (Table 1), as confirmed by methylation test and microsatellite analysis (data not shown).

All patients presented with classical PWS (cases 1–3 and 5) or AS (case 5) stigmata. Whole genome array-CGH analysis with commercially available (Agilent 244k and 180k) and high-resolution customized (eArray, covering the15q11-q13 region at 1 Kb resolution from 20.316 to 30.815 Mb, Agilent Technologies) platforms, were performed on all subjects in order to define the precise nature of each rearrangement and determine the boundaries of the 15q deletion and any additional microduplication/microdeletion of the chromosomes involved in each rearrangement.

**Figure 1:**
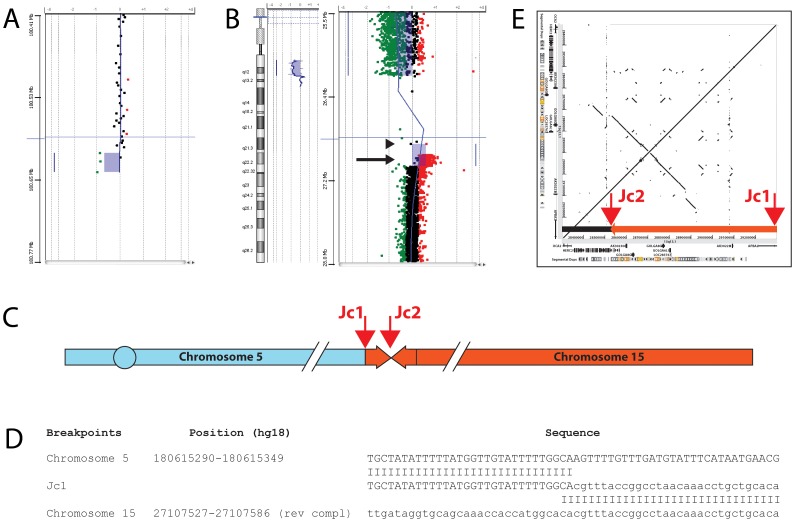
Molecular cloning of the 5;15 translocation in case 1. A, magnified view of the chromosome 5 breakpoint boundary detected by array-CGH using a 244 K oligonucleotide-based whole-genome microarray. The shaded area indicates a loss in DNA copy number (deletion) detected by three oligonucleotide probes (green dots). Black dots represent probes with no changes in copy number (non-deleted region). B, whole chromosome view (left) and magnified view (right) of the chromosome 15 breakpoint boundaries detected by custom oligonucleotide-based 15q11-q13 microarray. The shaded areas indicate a deletion (majority of green dots) and a gain in DNA copy number (duplication) detected by red dots (see arrow). The area containing few widely spaced probes represents BP3, a large region containing paralogous sequences. The last deleted oligomer is at 26,210,153 bp within *HERC2*, corresponding to BP3; the duplicated region is between 26,996,914 (first duplicated) and 27,106,557 bp (last duplicated) with first normal oligomer at 27,108,882 bp just distal to BP3, within the *APBA2* gene. An arrowhead points to the two black spots possibly indicating a single copy region between the deletion and the duplication. C, schematic representation of the rearrangement showing the two chromosomes involved, the position and orientation of the duplicated region, and the location of the two junctions (arrows). D, DNA sequences spanning the chromosome 5 deletion/15 duplication junction (Jc1) aligned with the reference sequences. E, dot-plot diagram, made with PipMaker software [45], showing the relative location of the inverted chromosome 15 duplication boundaries (Jc1 and Jc2, arrows) and of the *GOLGA8E*-associated inverted low copy repeat. The duplicated portion is represented by an orange arrow box.

In **case 1** (Case 2 in [11]), array-CGH analysis identified a 5q deletion of about 240 kb (fig. 1a), as well as a 100 kb duplication contiguous to the 15q deletion (fig. 1b). Using qPCR, we restricted the location of the chromosome 15 duplication breakpoints proximally to a 14 kb region inside the BP3 segmental duplication and distally to a 2 kb sequence (Table 1). We also restricted the chromosome 5 breakpoint to a 600 bp region. We successfully amplified the 5; distal 15 junction (Jc1) by LR-PCR (fig. 1d), demonstrating that the duplicated portion of chromosome 15 is inverted, as in most inv dup del rearrangements [12]. A 300 kb inverted repeat partially overlapping the duplicated region (fig. 1e) may be responsible for the genesis and location of the rearrangement. The chromosome 15 breakpoint is inside a LINE repeat and the junction shows a 2-bp microhomology. The proximal chromosome 15 breakpoint is contained inside the BP3 segmental duplication. The complex organization of this region did not allow us to fully characterize the junction (Jc2) and confirm the existence of the single-copy region suggested by array-CGH data (Fig. 1b, arrowhead). The duplication is not present in the database of genomic variants (http://projects.tcag.ca/variation/) and its *de novo*/inherited occurrence remains unknown because we did not have enough material from the parents to perform an array investigation.

**Case 2** (Case 3 in [13]; case 1 in [11]) is a “jumping translocation” with a major t(15;18) and a minor t(X,15) cell line. Both lines were present in peripheral blood and in fibroblasts. Array-CGH analysis confirmed the absence of any copy number change of the recipient chromosomes 18 (last oligo on 244 K platform: 76110964-76111023) and X (although the very low percentage of cells carrying the t(15;X) prevented a completely reliable estimate). Array-CGH and qPCR analysis identified the chromosome 15 breakpoint at 15q11.2 distal to SNRPN, between BP2 and BP3, within a 7.5 kb cluster of Alu and LINE repeats. We were not able to clone the breakpoint junction by either inverse PCR or Annealing Control Primer (ACP) -PCR.

In **case 3**, array-CGH analysis demonstrated that the recipient chromosome 6 carried no copy number changes (first oligo on the platform: 97634-97693). Array-CGH and qPCR analysis narrowed the location of the chromosome 15 breakpoint to a 585 bp region within the *OCA2* gene. Neither inverse PCR nor ACP-PCR allowed cloning of the breakpoint junction.

**Case 4** was the only one in which the translocation was not *de novo*, but inherited from the balanced mother. Array-CGH analysis showed that the size of the 9p deletion was about 4 Mb and placed the 15q12 breakpoint within the *ATP10A* gene, between BP2 and BP3. We amplified the 9;15 junction fragment by LR-PCR. The chromosome 9 breakpoint falls in a MIR repeat within intron 4 of the *GLIS3* gene, while the chromosome 15 breakpoint is in intron 1 of *ATP10A*. The junction shows only a 1-bp micro-homology.

**Case 5** was a mosaic with, at least in blood, a major cell line having 46 chromosomes and a reciprocal translocation t(8;15) and a minor one having 45 chromosomes and missing the der(15). Array-CGH with the 180 K platform, performed on DNA from blood, did not reveal any genomic abnormality whereas the same experiment on saliva DNA identified a 15q deletion with breakpoint in 15q13.3 within an about 8.9 kb region upstream of the *FMN1* gene. The log_2_ ratio values suggested a mosaic of about 30%, similar to what had been detected in blood by conventional cytogenetics (see Table 1).

Chromosome 8 did not show any evidence of copy number changes (first oligo on 180 K platform: 151472-151516). We were not able to restrict the 15q breakpoint by qPCR because of the low level of mosaicism.

## Discussion

### No clustering of the 15q breakpoints

**Figure 2:**
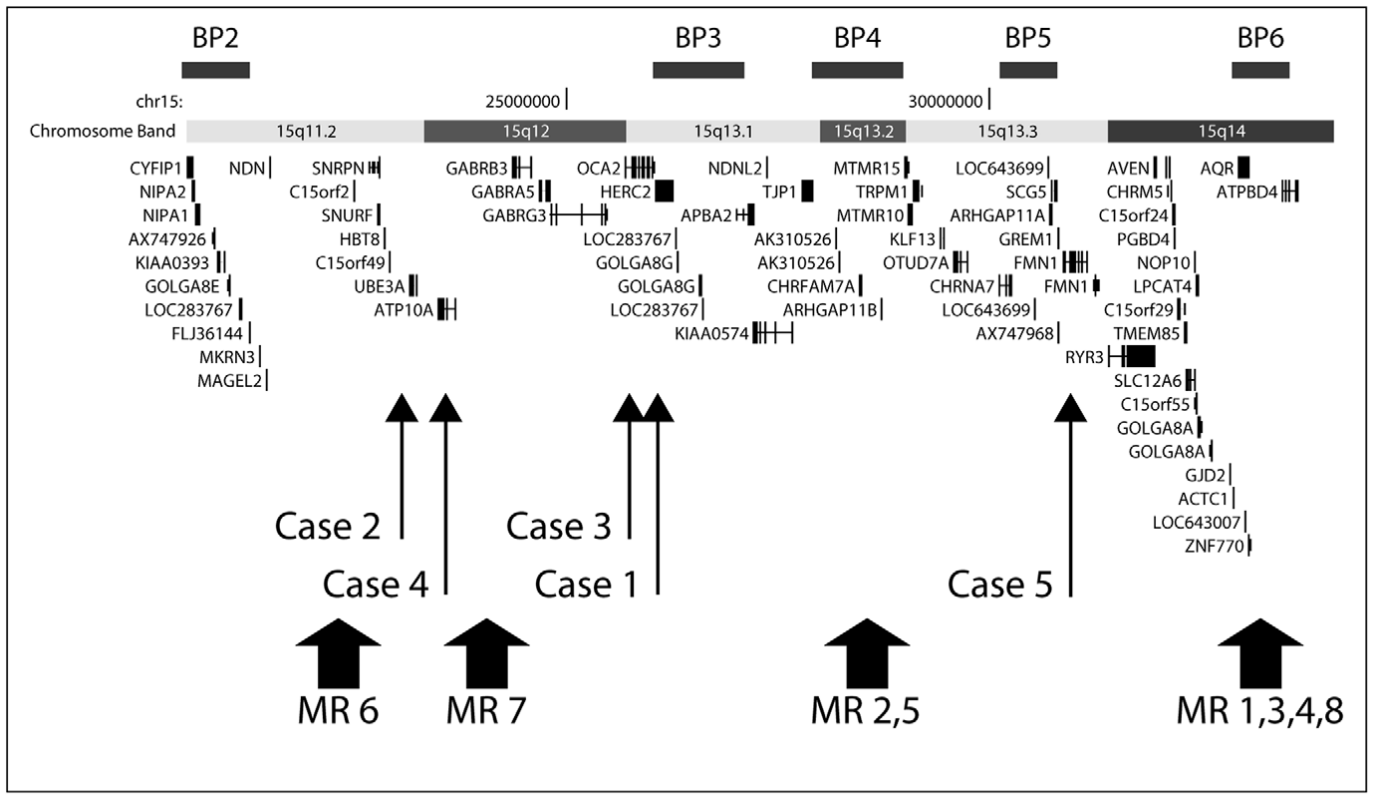
Physical map of the 15q11.2-q14 region. The six segmental duplication sites responsible for specific recurrent rearrangements in this region, known as BP1-6, are represented by black boxes. All genes in the region are shown. The position of the chromosome 15 breakpoints of the five translocation cases we have examined are represented by thin arrows. The positions of the eight translocation cases (MR1-8) described by Mignon-Ravix [10] are indicated by thick arrows.

Our data indicate that the 15q breakpoints of our unbalanced translocations fall within one of the segmental duplications in the region only in case 1 and that breakpoint locations were different in all cases. A map of the 15q11-q14 region, with the breakpoints of our cases and of cases with similar type of rearrangements studied by Mignon-Ravix et al [10], is shown in Fig. 2. In a total of 13 unbalanced translocations there is no breakpoint clustering, at least between BP2 and BP5. In the four cases reported by Mignon-Ravix et al [10] the claimed clustering at BP6 consisted of a 460 kb interval simply defined by FISH, and in any case outside the 15q segmental duplications.

### Mechanisms of formation

Since these translocations do not share recurrent breakpoints on chromosome 15, at first sight the mechanism of their formation should not have anything to do with non allelic homologous recombination (NAHR) as in the case, for example, of the recurrent t(4;8)(p16;p23) translocation in which the recombination occurs between one of two copies of homologous segmental duplications present both at 4p16 and 8p23 [14].

However, a casual occurrence of the 15q rearrangement is not very likely given the richness of the region in homologous segmental duplications, known to be responsible of recurrent deletions and duplications occurring by NAHR either between chromatids or chromosomes [3], [4].

**Figure 3:**
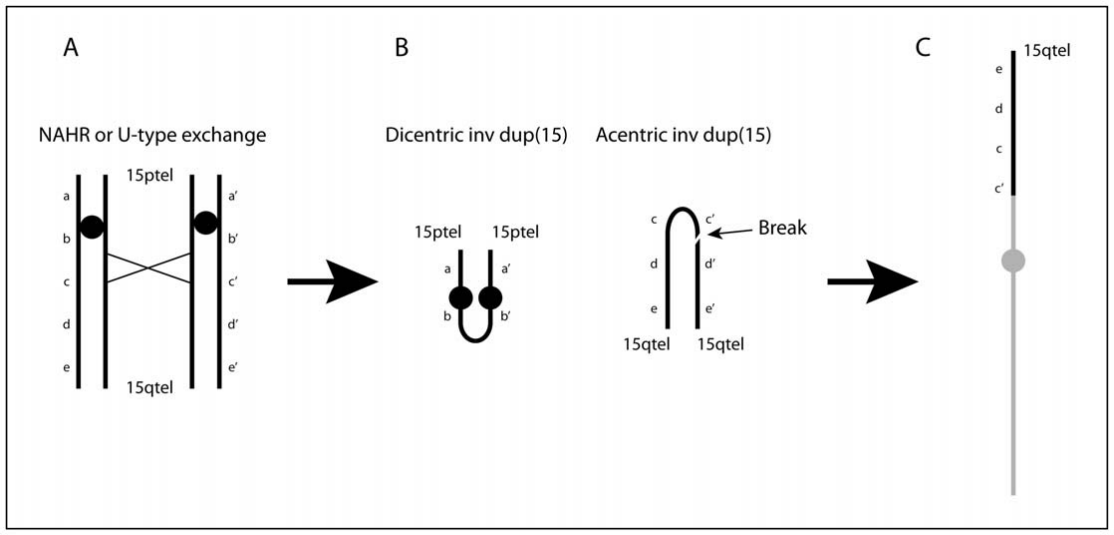
Schematic drawing of the putative mechanism leading to de novo unbalanced 15q translocations. (A) at meiosis, NAHR or U-type exchange, among others between LCRs BP3: BP3 or BP4: BP5, create (B) a mirror dicentric chromosome containing the p-arm and proximal q arm and a mirror acentric chromosome containing two copies of most of the q-arm. Rearrangements mediated by BP4: BP5 will generate dicentric and acentric chromosomes containing one copy of the sequence between the repeats including the PWS/AS region (not shown). The acentric/neocentric chromosome breaks, probably randomly, in two fragments of different size and (C) one of them attaches to the distal portion of a receiving chromosome (grey line). Attachment of the larger fragment containing an inverted duplicated portion, as in our Case 1, is depicted in the drawing.

Considering the mechanisms leading to the formation of supernumerary inverted duplicated marker chromosomes from chromosome 15, named “inv dup(15)s”, we hypothesized that the translocations could be the byproduct of the original 15qter->q1::q1->qter acentric chromosome reciprocal to the inv dup(15)s (Fig. 3).

inv dup(15)s can be small, clinically insignificant or larger, clinically important. These latter have been subdivided into two groups on the basis of their sizes, referred to as groups A and B. Group A inv dup(15)s are the “larger” ones which consists of two copies of the region 15pter to BP4 or BP5. Group B inv dup(15)s are the “smaller” ones with the dicentric chromosome mirrored around BP3 [15], [16]. In contrast to the group B inv dup(15)s that are always symmetrical BP3: BP3, group A inv dup(15)s are asymmetrical, one 15q arm of the inv dup(15) ends at BP4 and the other at BP5 (BP4: BP5) [17], [18]. BP4 and BP5 contain two large pairs of inverted segmental regions with low copy repeats, which facilitate genomic recombination between these two breakpoints. BP3 contains one smaller pair of inverted segmental repeats, facilitating BP3: BP3 recombination [17], [19], [20]. Recombination between BP4 and BP5 seems to be the predominant mechanism of formation of large inv dup(15)s that extend distal to BP3. Altogether, it has been assumed that inv dup(15)s may originate through different mechanisms: 1. Partial trisomy rescue, with the inv dup(15) being the early postzygotic consequence of the breakage of one of the three chromosomes 15 [21], [22] subsequently repaired by chromatid fusion. In this case, the marker should be a true isodicentric chromosome, as reported for some of them [23]; 2. NAHR between chromatids or chromosomes at meiosis I (Fig. 3A, B), with segregation into the final gamete of a normal chromosome 15 and the marker chromosome [24]. In this case, the phenotype of the subject carrying the supernumerary inv dup(15) will be normal or abnormal according to the size of the marker chromosome (presence/absence of the imprinted region); 3. NAHR between segmental duplications involving chromatids or chromosomes at meiosis I, with segregation into the final gamete of the marker chromosome alone followed by monosomy rescue of the chromosome 15 from the partner gamete [21]. In this case, the phenotype will be abnormal due to chromosome 15 UPD. In cases 2 and 3, the formation of the reciprocal product of an inv dup(15) (Fig. 3B) is consistent with reports that deletions of chromosome 15 associated with PWS/AS are also the result of inter- and intrachromosomal rearrangements [3], [4]. Considering the production of an inv dup(15) by NAHR, it seems likely that it will be complemented by the formation of a reciprocal 15qter->q1::q1->qter acentric product (Fig. 3B). Although this mechanism is well accepted for other recurrent rearrangements, such as the inv dup(8p)s where we demonstrated that NAHR between two segmental duplications at 8p23 produces a dicentric mirror chromosome 8qter->p23::p23->qter and its reciprocal acentric product 8pter->p23::p23->pter [25], the theoretical formation of a reciprocal acentric chromosome for the inv dup(15)s has never been taken into consideration.

However, there are examples in the literature showing that this mechanism actually occurs [26] and that the process of neocentromerization is quite frequent and leads to stable neocentric chromosomes [27], [28]. Thus it seems very likely that the reciprocal acentric product of the inv dup(15) has never been detected not because it does not contain any classical centromere but rather because a zygote with a normal chromosome 15 plus the whole 15qter->q1::q1->qter acentric or neocentric chromosome (from here on called the acentric/neocentric chromosome) would result in an almost complete trisomy 15. This situation is not compatible with embryo development unless at least partial trisomy rescue occurs by breakage and elimination of part of the acentric/neocentric chromosome. The trisomy 15 rescue should occur in the early embryo leading to different types of broken acentric/neocentric chromosomes, some of them maintained in the embryo itself because compatible with its development, others soon eliminated because containing too large or too small a portion of chromosome 15. Moreover, asymmetric breakage may result in a der(15)(q1->qter) with an inverted duplication and in a deleted der(15)(q1->qter) (Fig. 3C). In fact in our case 1, the rearranged chromosome 15, attached to the recipient chromosome 5, has a 100 Kb inverted duplication (Fig. 1B, C) derived from a symmetrical BP3: BP3 rearrangement akin to the one leading to Group B inv dup(15) formation. This byproduct of the original acentric/neocentric chromosome is then rescued by telomere capture leading to an unbalanced translocation of the type we are discussing. This mechanism would also explain the rearrangements in cases 2 and 3, assuming that the smaller non-duplicated asymmetric breakage product of the acentric/neocentric chromosome was preserved (Fig. 3C). In case 2, the translocation was “jumping” with a portion of 15q1-qter transposed to two different chromosomes in independent cell lines, suggesting that the acentric/neocentric chromosome had been preserved for a given, presumably short, period in embryogenesis. The finding that other jumping translocations have been reported with apparently the same portion of 15q1 translocated to up to three or even four different recipient chromosomes [29], [30] might reflect the persistence of an acentric/neocentric chromosome breaking down independently in separate early embryo cells and donating slightly dissimilar portions to different chromosomes. In this light, jumping translocations should not be considered as the result of the transposition of the same chromosomal portion to different chromosomes in different cells but rather as the result of distinct events of trisomy rescue. In fact, independent rescue events of an original trisomy have been reported leading to mosaic situation with a normal cell line and a second one with UPD for the originally trisomic chromosome [31].

Accordingly to the mechanism of formation of the unbalanced translocations we are discussing, we should expect that in some cases both products of the 15q1 meiotic NAHR between segmental duplications would co-segregate to the same gamete leading, in the early embryo, to cells containing both an unbalanced translocation and an inv dup(15). This has in fact been reported in few cases [11], [32].

Against this hypothesis is the finding that inv dup(15)s are quite frequent (0.044% in newborns: www.fish.uniklinikum-jena.de/sinv dup/index.html; about 60% of all supernumerary marker chromosomes: Blennow et al. [33]) whereas the 45 chromosomes unbalanced translocations are rare. This likely depends on the presumably high lethality of embryos with 45 chromosomes plus a mirror acentric/neocentric chromosome. Moreover, most inv dup(15)s associated with a phenotype are of maternal origin [34], [35] whereas in our hypothesis the rearrangement resulting in inv dup(15) and its reciprocal mirror acentric chromosome should occur at both maternal and paternal meiosis as demonstrated by the phenotype of the unbalanced translocation carriers, that can be either AS or PWS. It is possible that some form of selection against the larger inv dup(15)s occurs during spermatogenesis as it has in fact been shown by sperm analysis studies of males carrying the smaller inv dup(15)s [36] so that maternal oocytes with the supernumerary inv dup(15) may be more likely to complete gametogenesis.

The mechanism of formation of the unbalanced translocations in cases 4 and 5 does not fit the previously proposed model. Cases 4 and 5 appear to be classical translocations, with the der(15) lost at maternal meiosis as a consequence of a 3∶1 segregation in case 4, and with the der(15) lost during embryogenesis in part of the cells in case 5. In both cases, the der(15) is the reciprocal product of a balanced translocation, not the supernumerary inv dup(15) we postulated as the reciprocal product of our *de novo* unbalanced translocations.

### Conclusions

The hypothesis that some unbalanced 45 chromosomes translocations derive from a two-step mechanism with a meiotic start and a postzygotic re-adjustment is not at all new. This mechanism has been largely demonstrated to form the basis of several inv dup del rearrangements, both recurrent and sporadic, including a few unbalanced translocations [12], [25]. In the case of the recurrent inv dup del(8p), we have demonstrated by microsatellite analysis that the dicentric mirror chromosome originated at maternal meiosis I as a consequence of NAHR, and underwent postzygotic breakage leading to an inv dup del(8p) with duplications of different size in different subjects.

Also for this rearrangement, some cases have been detected [37], [38] with more than one cell line carrying different portions of the original dicentric chromosome in the early embryo. Moreover, we also demonstrated that the reciprocal product of the dicentric chromosome is a neocentric chromosome 8(pter->p23.1::p23.1->pter). Thus the mechanisms of rearrangement by NAHR at 8p23 and 15q1 are quite similar, the only difference being the size of the two reciprocal products: the original 8qter->p23::p23->qter dicentric chromosome is large and undergoes successive breakage while the acentric/neocentric 8pter->p23::p23->pter chromosome is small and its preservation as a supernumerary marker is compatible with embryo development; on the contrary, the dicentric inv dup(15) is small while the 15qter->q1::q1-qter acentric/neocentric is large and needs to be partly deleted to insure embryo development.

Our hypothesis is based on the assumption that a neocentromere would initially stabilize the large acentric fragment, thus impairing its loss. This hypothesis is not unsubstantiated. First of all, a number of neocentromeres have been reported in pre- and postnatal life, often arising on the distal 15q [28], [39], [40] and are a frequent finding in cancer [39] thus demonstrating that their occurrence can be a quick solution providing a reproductive advantage to the cell. Moreover, several neocentromeres are evolutionary and do not have any phenotypic consequence [41]. These evolutionary neocentromeres fixed in the population represent only the tip of the iceberg but demonstrate the frequent occurrence of this mechanism.

Murmann et al [27], on the basis of the identification of DNA sequence motifs with inverted homologies within breakpoint regions in 12 acentric marker chromosomes, proposed that an acentric fragment would form following a double-strand break during either meiosis or mitosis; the fragment would be stabilized by the formation of an intra-chromosomal loop promoted by the presence of sequences with inverted homologies. In this respect, it is also worth noting that Zeitlin et al. [42] have documented the involvement of CENP-A in DNA repair. It can be hypothesized that the DNA damage *per se* could trigger neocentromere formation. This stabilized fragment would be duplicated during an early mitotic event, likely in coincidence with neocentromere formation, insuring the marker's survival during cell division and its presence in all cells. This interesting mechanism does not rule out the one we are proposing here. However, we also include the possibility that acentric and reciprocal dicentric chromosomes may also have occurred through interchromosomal exchange as we demonstrated for the inv dup (8p) and, as a consequence, for its reciprocal acentric product. Moreover our hypothesis is also in agreement with the finding that 15q11-q13 deletions in PWS and AS cases may occur both through inter- and intrachromosomal exchanges [3], [4].

## Materials and Methods

### Ethics Statement

This study has been approved by the Ethics Committee at the University of Pavia.

### Array-CGH studies

A customized array-CGH platform was generated using eArray software (http://earray.chem.agilent.com); the probes (60 mer oligonucleotides) were selected from those available in the Agilent database. The array was made by 43100 probes, of which 10534 represented the 15q11-q13 chromosome region between 20.316 Mb and 30.815 Mb (UCSC, hg18), allowing molecular profiling of 15q11-q13 breakpoints with a resolution of about 1 Kb. Genomic DNA was isolated from blood or saliva samples using the GenElute-Blood kit (Sigma). Gender-matched genomic DNAs were obtained from individuals NA10851 (male) and NA15510 (female) (Coriell). The quality of each DNA was evaluated by conventional absorbance measurements (NanoDrop 1000, Thermo Scientific) and electrophoretic gel mobility assays. Quality of experiments was assessed using Feature Extraction QC Metric v10.1.1 (Agilent). The derivative log ratio spread (DLR) value was calculated using the Agilent Genomics Workbench software. Only experiments having a DLR spread value <0.30 were taken into consideration.

### qPCR

We used quantitative PCR (qPCR) to verify and restrict the breakpoint regions characterized by aCGH. All amplicons were chosen within non-repeated portions of the chromosome using Primer Express 3.0 software (Applied Biosystems, Foster City, CA, USA); a control amplicon was selected with the same parameters in the *MAPK1* gene on 22q11.2; size (approximately 60 bp) and Tm (58°C) were the same for all amplicons. Sequence coordinates are shown according to the UCSC Human Genome Browser, Database hg18, March 2006 Assembly (genome.ucsc.edu/cgi-bin/hgGateway). We performed amplification and detection on a ABI PRISM 7900 Sequence Detection System (Applied Biosystems) using SYBR Green PCR Master Mix (Applied Biosystems); thermal cycling conditions were 50°C for 2 min and 95°C for 10 min, followed by 40 cycles of 95°C for 15 sec and 60°C for 1 min; all samples were amplified in duplicate. Validation experiments demonstrated that amplification efficiencies of the control and target amplicons were approximately equal; accordingly, relative quantification of the amount of DNA was obtained using the comparative CT method [43].

### Long-Range PCR (LR-PCR) and sequencing

We performed long-range PCRs with JumpStart Red ACCUTaq LA DNA polymerase (Sigma) and the following protocol: 30 sec at 96°C; 35 cycles of 15 sec at 94°C/20 sec at 58°C/15 min at 68°C; 15 min final elongation time. Sequencing reactions were performed with a Big Dye Terminator Cycle Sequencing kit (Applied Biosystems) and run on an ABI Prism 3130xl Genetic Analyzer. (Primer sequences are available on request).

### Methylation-specific PCR

Methylation analysis was performed in case 1–4, with DNA modified by bisulphate treatment and *SNRPN* exon 1 amplified by PCR, according to standard protocols [44].

### Genotyping

Genotyping of polymorphic loci was performed in case 5 by amplification with primers labelled with fluorescent probes (ABI 5-Fam and Hex) followed by analysis on an ABI 310 Genetic Analyzer (Applied Biosystems). The UCSC Genome Browser maps and sequences were used as references. Amplification was performed with Taq Gold (Applied Biosystems) using standard protocols. The following polymorphic markers in the deleted region were used: D15S1035, D15S817, D15S822, D15S1002, D15S986 and D15S1021.
